# Causal association between constipation and risk of colorectal cancer: a bidirectional two-sample Mendelian randomization study

**DOI:** 10.3389/fonc.2023.1282066

**Published:** 2023-11-16

**Authors:** Long Wu, Huan Wu, Fei Huang, Xiao-yun Li, Yun-huan Zhen, Bao-fang Zhang, Hai-yang Li

**Affiliations:** ^1^ Department of Anus and Intestinal Surgery, The Affiliated Hospital of Guizhou Medical University, Guiyang, Guizhou, China; ^2^ Department of Infectious Diseases, The Affiliated Hospital of Guizhou Medical University, Guiyang, Guizhou, China; ^3^ Department of Hepatobiliary Surgery, The Affiliated Hospital of Guizhou Medical University, Guiyang, Guizhou, China

**Keywords:** constipation, colon cancer, rectal cancer, colorectal cancer, Mendelian randomization

## Abstract

**Background:**

Colorectal cancer (CRC) is a globally significant health concern, necessitating effective preventive strategies through identifying modifiable risk factors. Constipation, characterized by infrequent bowel movements or difficulty passing stools, has been proposed as a potential CRC risk factor. However, establishing causal links between constipation and CRC remains challenging due to observational study limitations.

**Methods:**

Mendelian randomization (MR) utilizes genetic variants as instrumental variables, capitalizing on genetically determined variation to assess causal relationships. In this dual-sample bidirectional MR study, we extracted genetic data from independent cohorts with CRC (Include colon cancer and rectal cancer) and constipation cases. Genome-wide association studies (GWAS) identified constipation and CRC-associated genetic variants used as instruments to infer causality. The bidirectional MR analysis evaluated constipation’s impact on CRC risk and the possibility of reverse causation.

**Results:**

Employing bidirectional MR, we explored the causal relationship between constipation and CRC using publicly available GWAS data. Analysis of constipation’s effect on CRC identified 26 significant SNPs, all with strong instrumental validity. IVW-random effect analysis suggested a potential causal link [*OR* = 1.002(1.000, 1.004); *P* = 0.023], although alternative MR approaches were inconclusive. Investigating CRC’s impact on constipation, 28 significant SNPs were identified, yet IVW analyses found no causal effect [*OR* = 0.137(0.007, 2.824); *P* = 0.198]. Other MR methods also yielded no significant causal association. We analyzed constipation separately from colon and rectal cancer using the same methodology in both directions, and no causal relationship was obtained.

**Conclusion:**

Our bidirectional MR study suggests a potential constipation-CRC link, with mixed MR approach outcomes. Limited evidence supports constipation causing CRC. Reliable instruments, minimal heterogeneity, and robust analyses bolster these findings, enriching understanding. Future research should explore additional factors to enhance comprehension and clinical implications.

## 1 Introduction

Colorectal cancer (CRC) stands as a global health concern, contributing significantly to morbidity and mortality rates worldwide (1, 2). The quest to identify modifiable risk factors for CRC is of paramount importance to develop effective preventive strategies (3, 4). Among the potential risk factors, constipation, characterized by infrequent bowel movements or difficulty passing stools, has garnered attention for its potential association with CRC risk (5, 6). Previous observational studies have generated substantial controversy regarding the causal relationship between constipation and CRC (7–11). Establishing a definitive causal relationship between constipation and CRC remains challenging due to inherent limitations within observational studies (12).

To address these challenges, we present a pioneering approach that employs Mendelian randomization (MR) analysis to investigate the potential causal link between constipation and CRC risk (13). MR leverages genetic variants, often using single nucleotide polymorphisms(SNPs) as instrumental variables, utilizing their strong associations with constipation to infer causality (14). This approach offers a unique advantage by minimizing biases arising from confounding and reverse causation, which often impede the accuracy of observational studies (13, 15).

Our research framework involves the use of large-scale genetic and epidemiological datasets to perform a bidirectional MR analysis. This analysis investigates both the effect of constipation on CRC risk and the reciprocal influence of CRC risk on constipation occurrence. By elucidating these bidirectional relationships, we aim to provide robust evidence that contributes to our understanding of the interplay between constipation and CRC.

## 2 Methods

### 2.1 Data sources and selection of genetic variants

**Figure 1:**
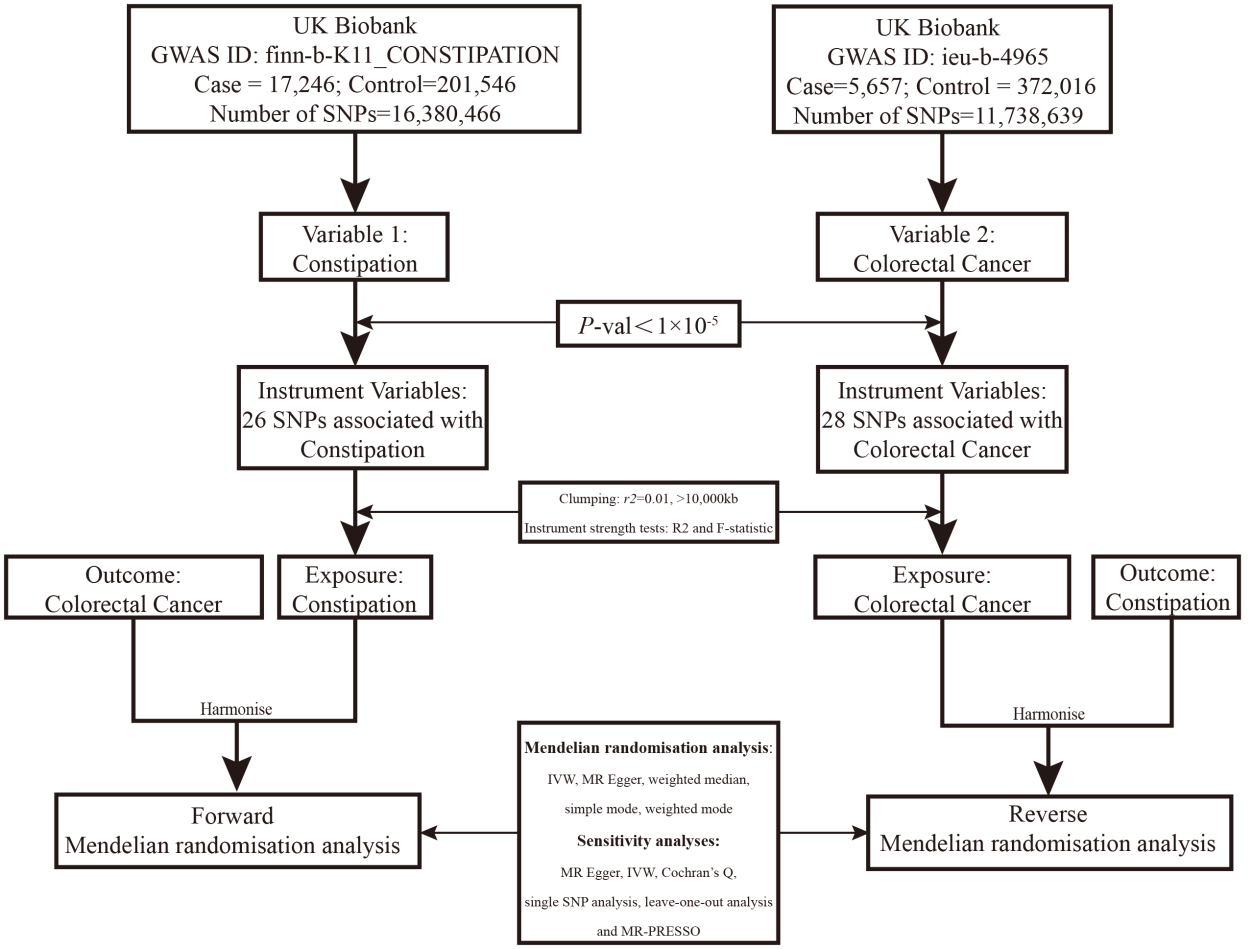
Overview of the two-sample MR study design used to investigate the causal association between constipation and CRC.

We searched the Open Genome-Wide Association Studies (OpenGWAS) database for curated GWAS summary datasets. Using publicly available data, we conducted a two-sample MR study, using genetic variants linked to constipation and colorectal cancer as instrumental variables (IVs) with a *P*-value threshold of 1×10^-5^ to get enough SNPs, and pruned IVs by linkage disequilibrium (LD) (r^2^≥0.01, kb>10,000) (16). In addition, palindromic SNPs were removed by using minor allele frequencies to prevent strand ambiguity issues (17). We computed the R^2^ statistic and F statistic for the instrumental variables in the exposure. The R^2^ statistic signifies the variance explained by the instrumental variables. Each individual variant demonstrated an F statistic equal to or exceeding 10, indicating strong instrumental variables. An F statistic below 10 is generally deemed a ‘weak IV’. Hence, the potential for weak instrument bias in our analysis was notably low. Constipation data from European individuals (*n* = 218,810), colorectal cancer data from Europeans (*n* = 377,673), colon cancer (CC) data from Europeans (*n* = 462,933) and rectal cancer (RC) data from Europeans (*n* = 456,276) were used. Data details can be found at https://gwas.mrcieu.ac.uk/. See **Figure 1** for the analysis flow.

Moreover, we used PhenoScanner (http://www.phenoscanner.medschl.cam.ac.uk/) to check if instruments used were associated with potential confounders for the effect of constipation on CRC, and of CRC on constipation. We performed a leave-one-out analysis to check if any individual SNP was driving the observed association for both constipation on CRC, and for CRC on constipation.

### 2.2 Mendelian randomization analysis

This study utilized the Inverse Variance Weighted (IVW) - Random Effects as the primary MR method. Four additional MR approaches were employed: Weighted Median, Weighted Mode, Simple Mode, and MR-Egger. IVW involves meta-analyzing SNP exposure and outcome effects, adjusting for heterogeneity. The Weighted Median calculates the median causal estimate, Weighted Mode identifies the mode, and Simple Mode estimates causality without weights. MR-Egger addresses pleiotropy. Combining methods enhances robustness, offering varied insights. IVW assumes valid instrumental variables; deviations impact precision. These techniques provide a comprehensive view of the causal relationship, considering different assumptions and biases. All Mendelian randomization analyses were conducted using the RStudio Software (Version: 2023.06.0 Build 421) and R Software (Version: 4.3.1).

### 2.3 Heterogeneity and sensitivity analysis

We examined the heterogeneity between SNPs using Cochran’s Q-statistics (18) and I^2^ statistic (19, 20). Additionally, we conducted a “leave-one-out” analysis to explore the potential influence of individual SNPs on the causal association (21).

## 3 Results

### 3.1 Effect of constipation on CRC

**Figure 2:**
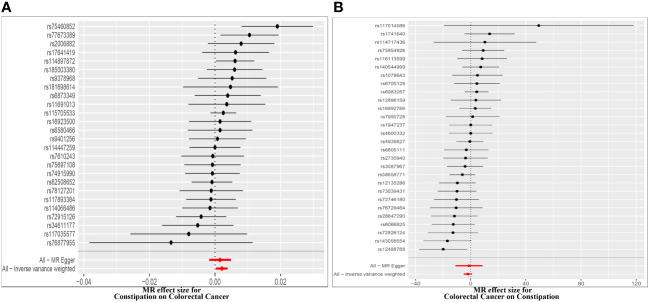
Forest plot of MR effect of the causal relationship between constipation and CRC **(A)**. Effect of Constipation on CRC; **(B)** Effect of CRC on Constipation.

**Figure 3:**
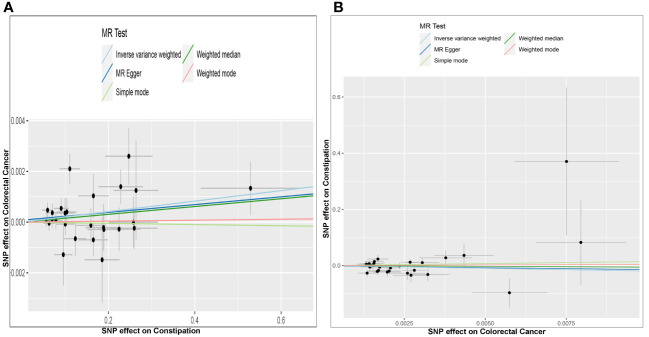
Scatter plots of genetic associations between constipation and CRC. The slopes of each line represent the causal association for each method. The light blue line represents the inverse‐variance weighted estimate, the green line represents the weighted median estimate, the dark blue line represents the Mendelian randomization‐Egger estimate, the red line represents the weighted mode estimate, and the light green line represents the simple mode estimate **(A)**. Effect of Constipation on CRC; **(B)** Effect of CRC on Constipation.

Effect of Constipation on CRC: The Constipation GWAS identified 26 independent genome-wide significant SNPs. All SNPs utilized in the MR analysis were considered “strong” instruments, each possessing an F statistic greater than 10. The F statistic takes into account the SNP’s effect magnitude and precision on Constipation. Individual F statistics ranged from 20 to 30. While IVW-random effect analysis indicated a potential causal link between Constipation and CRC odds [*OR* = 1.002 (1.000, 1.004); *P* = 0.023], the other four approaches did not provide substantial evidence of a causal association (**Table 1**, **Figure 2**, **Figure 3**).

**Table 1:** Results of two-sample bidirectional MR analysis of the causal effects between Constipation and CRC (include CC and RC).

Exposures	Outcomes	Methods	Number of SNPs	*Beta*	*SE*	*P-vale*	*OR*	*95%CI*
Constipation	CRC	MR Egger	26	0.002	0.002	0.371	1.002	0.998, 1.005
		Weighted median	26	0.002	0.001	0.213	1.002	0.999, 1.004
		Inverse variance weighted	26	0.002	0.001	0.023	1.002	1.000, 1.004
		Simple mode	26	0.000	0.002	0.913	1.000	0.996, 1.004
		Weighted mode	26	0.000	0.002	0.915	1.000	0.997, 1.004
	CC	MR Egger	4	0.024	0.022	0.392	1.024	0.981, 1.069
		Weighted median	4	0.002	0.001	0.155	1.002	0.999, 1.004
		Inverse variance weighted	4	0.002	0.001	0.086	1.002	1.000, 1.004
		Simple mode	4	0.002	0.002	0.380	1.002	0.998, 1.006
		Weighted mode	4	0.002	0.002	0.370	1.002	0.998, 1.005
	RC	MR Egger	3	3.574	1.626	0.272	35.649	1.472, 863.106
		Weighted median	3	-0.157	0.448	0.726	0.855	0.355, 2.057
		Inverse variance weighted	3	-0.194	0.616	0.753	0.824	0.246, 2.756
		Simple mode	3	1.420	1.272	0.380	4.137	0.342, 50.041
		Weighted mode	3	-0.657	0.407	0.248	0.518	0.233, 1.151
CRC	Constipation	MR Egger	28	-1.271	4.987	0.801	0.281	0.000, 4932.099
		Weighted median	28	-0.564	2.087	0.787	0.569	0.010, 34.019
		Inverse variance weighted	28	-1.990	1.545	0.198	0.137	0.007, 2.824
		Simple mode	28	1.403	4.192	0.740	4.067	0.001, 15052.811
		Weighted mode	28	0.401	3.32	0.905	1.493	0.002, 1000.545
CC		MR Egger	7	-1.719	180.121	0.993	0.179	8.540E-155, 3.770E+152
		Weighted median	7	16.285	11.736	0.165	1.181E07	0.001, 1.153E+17
		Inverse variance weighted	7	13.553	9.902	0.171	7.693E05	0.003, 2.063E+14
		Simple mode	7	22.757	20.004	0.299	7.643E09	7.160E-08, 8.160E+26
		Weighted mode	7	21.071	18.778	0.305	1.416E09	1.470E-07, 1.370E+25
RC		MR Egger	46	0.08	3.892	0.984	1.083	0.001, 2227.158
		Weighted median	46	-0.266	0.14	0.057	0.766	0.583, 1.008
		Inverse variance weighted	46	-0.235	0.121	0.053	0.791	0.623, 1.003
		Simple mode	46	-0.279	0.322	0.391	0.757	0.403, 1.422
		Weighted mode	46	-0.275	0.316	0.387	0.759	0.409, 1.109

MR, Mendelian Randomization; CRC, Colorectal Cancer; CC, Colon Cancer; RC, Rectal Cancer; SNP, Single Nucleotide Polymorphism.

Effect of Constipation on CC: The Constipation GWAS identified 4 independent genome-wide significant SNPs. All SNPs utilized in the MR analysis were considered “strong” instruments, each possessing an F statistic greater than 10. The F statistic takes into account the SNP’s effect magnitude and precision on Constipation. All the five approaches did not provide substantial evidence of a causal association (All results in **Table 1**).

Effect of Constipation on RC: The Constipation GWAS identified 3 independent genome-wide significant SNPs. All SNPs utilized in the MR analysis were considered “strong” instruments, each possessing an F statistic greater than 10. The F statistic takes into account the SNP’s effect magnitude and precision on Constipation. All the five approaches did not provide substantial evidence of a causal association (All results in **Table 1**).

### 3.2 Effect of CRC on constipation

Effect of CRC on Constipation: The GWAS on CRC identified 28 independent genome-wide significant SNPs. All SNPs used in the MR analysis were “strong” instruments with an F statistic >10, where the F statistic is a function of both magnitude and precision of the SNP’s effect on Constipation. Individual F statistics ranged from 21 to 90. The IVW-random effect analyses showed no evidence of a causal effect of CRC on the odds of Constipation [*OR* =0.137 (0.007, 2.824); *P* = 0.198]. In addition, other four approaches did not yield evidence of a causal association of CRC on the odds of Constipation (**Table 1**, **Figure 2**, **Figure 3**).

Effect of CC on Constipation: The GWAS on CC identified 28 independent genome-wide significant SNPs. All SNPs used in the MR analysis were “strong” instruments with an F statistic >10, where the F statistic is a function of both magnitude and precision of the SNP’s effect on Constipation. All the five approaches did not yield evidence of a causal association of CC on the odds of Constipation (**Table 1**).

Effect of RC on Constipation: The GWAS on RC identified 46 independent genome-wide significant SNPs. All SNPs used in the MR analysis were “strong” instruments with an F statistic >10, where the F statistic is a function of both magnitude and precision of the SNP’s effect on Constipation. All the five approaches did not yield evidence of a causal association of RC on the odds of Constipation (**Table 1**).

### 3.3 Heterogeneity and sensitivity analysis

**Figure 4:**
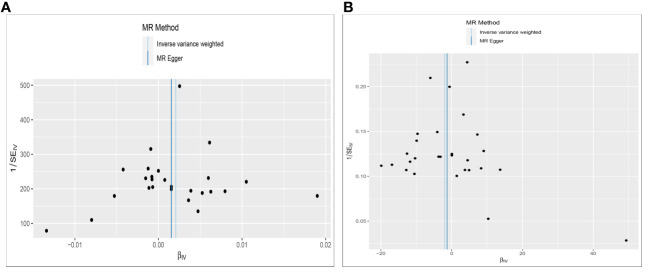
Funnel plot to assess heterogeneity. The light blue line represents the inverse‐variance weighted estimate, and the dark blue line represents the Mendelian randomization‐Egger estimate **(A)**. Effect of Constipation on CRC; **(B)** Effect of CRC on Constipation.

**Figure 5:**
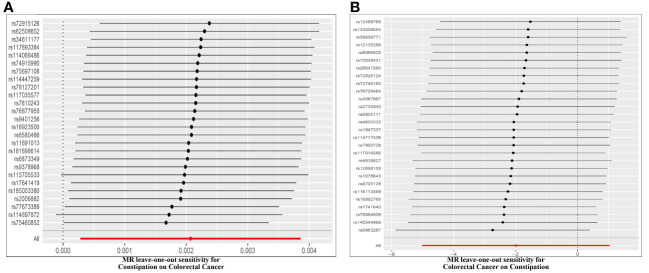
Leave-one-out of SNPs associated with constipation and CRC. Each black point represents result of the IVW MR method applied to estimate the causal effect between constipation and CRC excluding particular SNP **(A)**. Effect of Constipation on CRC; **(B)** Effect of CRC on Constipation.

Cochran’s Q test assessed heterogeneity among instrumental variable estimates from individual genetic variants. The results showed no significant evidence of heterogeneity (**Table 2**, **Figure 4**). Low heterogeneity suggests more reliable Mendelian randomization (MR) estimates. I^2^ values also indicated low heterogeneity, reinforcing MR estimate reliability (**Table 2**). “Leave-one-out” analysis, where each SNP was removed to assess its impact on the IVW point estimate (**Figure 4**), indicated no single SNP significantly influenced the overall result. The funnel plot and MR Egger regression test displayed no significant asymmetry, indicating minimal publication bias and directional horizontal pleiotropy (**Figure 5**). Overall, minimal heterogeneity, low I^2^ values, stable “leave-one-out” results, and absence of asymmetry confirm MR estimate reliability and mitigate bias concerns.

**Table 2:** the results of heterogeneity and sensitivity test.

MR analysis	Methods	Heterogeneity test				Sensitivity test		
-	-	*Q*	*df*	*Q-val*	*I^2^*	Egger regression intercept	Standard error	Directionality *P*-value
Constipation on CRC	MR Egger	29.223	24	0.212	0.150	7.680E-05	2.000E-4	0.707
	Inverse variance weighted	29.399	25	0.248	0.179			
Constipation on CC	MR Egger	0.607	2	0.738	2.294	-0.001	0.001	0.423
	Inverse variance weighted	1.606	3	0.658	0.868			
Constipation on RC	MR Egger	0.034	1	0.854	28.439	-2.197	0.924	0.253
	Inverse variance weighted	5.688	2	0.058	0.648			
CRC on Constipation	MR Egger	3.443	7	0.841	0.012	-0.002	0.010	0.880
	Inverse variance weighted	8.097	8	0.424	1.033			
CC on Constipation	MR Egger	8.773	5	0.118	0.430	0.009	0.105	0.936
	Inverse variance weighted	8.785	6	0.186	0.317			
RC on Constipation	MR Egger	4.112	44	1	9.700	-0.048	0.590	0.936
	Inverse variance weighted	4.119	45	1	9.926			

MR, Mendelian Randomization; CRC, Colorectal Cancer; CC, Colon Cancer; RC, Rectal Cancer.

## 4 Discussion

The findings of our bidirectional MR study provide valuable insights into the relationship between constipation and the risk of developing CRC. Our results suggest that constipation is associated with an increased risk of CRC, indicating a potential role for constipation as a modifiable risk factor for CRC. The observed link between constipation and an increased risk of CRC aligns with previous epidemiological and clinical studies (22), but not with the results of systematic review (7). Chronic constipation may lead to prolonged exposure to potential carcinogens in the colon, such as bile acids, which can promote tumor growth and initiate colorectal cancer (23). Additionally, constipation can disrupt the gut microbiota composition and function, leading to dysbiosis, increased inflammation, and altered metabolism of dietary components, all of which have been implicated in CRC development (12). Although our analyses found a causal relationship between constipation and colorectal cancer, this relationship was not statistically strong, and when constipation was analyzed separately from colon and rectal cancer, no such relationship was found. The possible reason for this may be that there are other potential risk factors that may interact with constipation and CRC.

The lack of evidence supporting a reverse causation relationship, where CRC would increase the risk of constipation, is an interesting finding. Some previous observational studies have suggested that there is no bidirectional relationship between CRC and constipation (7), our bidirectional MR analysis did not support this view. This finding suggests that CRC development may not directly contribute to the occurrence of constipation, but rather highlights the potential impact of constipation as a risk factor for CRC.

The strengths of our study lie in its utilization of a bidirectional MR approach, which provides stronger evidence for causal relationships compared to traditional observational studies. By leveraging SNPs as instrumental variables, we effectively address the issue of reverse causation and minimize the impact of confounding factors (15). Additionally, the use of two large, independent cohorts enhances the robustness and generalizability of our findings (13). However, several limitations should be considered when interpreting our results. Firstly, the bidirectional MR approach assumes that the instrumental variables are valid and accurately represent the exposure of interest. Although we carefully selected genetic variants associated with constipation and CRC from GWAS, the possibility of pleiotropy, where the genetic variants influence other pathways apart from constipation or CRC, cannot be entirely ruled out. Secondly, our study primarily focuses on the genetic predisposition to constipation and CRC and does not account for potential environmental or lifestyle factors that may mediate the observed associations. Future research should explore the potential mechanisms underlying the association between constipation and CRC to better understand the biological pathways involved. Additionally, efforts should be directed toward investigating other potential risk factors that may interact with constipation and CRC, such as dietary habits, physical activity, and medication use.

In conclusion, our bidirectional MR study provides evidence supporting the hypothesis that constipation increases the risk of developing CRC. These findings highlight the importance of managing constipation as a potential modifiable risk factor for CRC. Public health interventions should focus on promoting regular bowel movements, healthy dietary habits, and maintaining a balanced gut microbiota to reduce the risk of CRC in individuals with a history of constipation. Further research is warranted to validate our findings and explore additional factors associated with constipation and CRC risk.

## Data availability statement

The datasets supporting the conclusions of this article are available in OpenGWAS database website (https://gwas.mrcieu.ac.uk/). The other data generated or analyzed during this study are available in this published article and its supplementary information files.

## Author contributions

LW: Conceptualization, Data curation, Formal Analysis, Funding acquisition, Investigation, Methodology, Project administration, Resources, Writing – original draft, Writing – review & editing. HW: Conceptualization, Data curation, Formal Analysis, Investigation, Methodology, Project administration, Software, Writing – original draft, Writing – review & editing. FH: Conceptualization, Data curation, Investigation, Methodology, Writing – original draft, Writing – review & editing. X-YL: Investigation, Methodology, Writing – original draft, Writing – review & editing. Y-HZ: Conceptualization, Funding acquisition, Investigation, Project administration, Supervision, Validation, Visualization, Writing – original draft, Writing – review & editing. B-FZ: Funding acquisition, Project administration, Supervision, Validation, Writing – original draft, Writing – review & editing. H-YL: Investigation, Project administration, Supervision, Validation, Visualization, Writing – original draft, Writing – review & editing.
